# Is single-child family associated with cardio-metabolic risk factors: the CASPIAN-V study

**DOI:** 10.1186/s12872-018-0844-y

**Published:** 2018-06-04

**Authors:** Roya Kelishadi, Mostafa Qorbani, Fatemeh Rezaei, Mohammad Esmaeil Motlagh, Shirin Djalalinia, Hasan Ziaodini, Majzoubeh Taheri, Fatemeh Ochi, Gita Shafiee, Tahereh Aminaei, Armita Mahdavi Gorabi, Ramin Heshmat

**Affiliations:** 1 Pediatrics Department, Child Growth and Development Research Center, Research Institute for Primordial Prevention of Non-communicable Disease, Isfahan University of Medical Sciences, Isfahan, Iran; 2 Non-Communicable Diseases Research Center, Alborz University of Medical Sciences, Karaj, Iran; 3 Chronic Diseases Research Center, Endocrinology and Metabolism Population Sciences Institute, Tehran University of Medical Sciences, Tehran, Iran; 4 Department of Social Medicine, Medical School, Jahrom University of Medical Sciences, Jahrom, Iran; 5 Pediatrics Department, Ahvaz Jundishapur University of Medical Sciences, Ahvaz, Iran; 6 Deputy of Research and Technology, Ministry of Health and Medical Education, Tehran, Iran; 7 Bureau of Health and Fitness, Ministry of Education and Training, Tehran, Iran; 8 Office of Adolescents and School Health, Ministry of Health and Medical Education, Tehran, Iran; 9 Student Research Committee, Alborz University of Medical Sciences, Karaj, Iran; 10 Department of Basic and Clinical Research, Tehran Heart Center, Tehran University of Medical Sciences, Tehran, Iran; 11 Endocrinology and Metabolism Research Center, Endocrinology and Metabolism Clinical Sciences Institute, Tehran University of Medical Sciences, Tehran, Iran

**Keywords:** Family dimension, Single-child family, Cardio-metabolic risk factors, Children, Adolescents

## Abstract

**Background:**

In the present study, the association of the cardio-metabolic risk factors and the status of single-child family were studied in a national representative sample of Iranian children and adolescents.

**Methods:**

This cross sectional study was conducted as the fifth round of “Childhood and Adolescence Surveillance and PreventIon of Adult Non- communicable disease” surveys. The students’ questionnaire was derived from the World Health Organization-Global School Student Health Survey. Using survey data analysis methods, data from questionnaires’; anthropometric measures and biochemical information analyzed by logistic regression analysis.

**Results:**

Overall, 14,274 students completed the survey (participation rate: 99%); the participation rate for blood sampling from students was 91.5%. Although in univariate logistic regression model, single child students had an increased risk of abdominal obesity [OR: 1.37; 95% CI: 1.19–1.58)], high SBP [OR: 1.58; 95% CI:1.17–2.14)], high BP [OR: 1.21; 95% CI:1.01–1.45)] and generalized obesity [OR: 1.27; 95% CI:1.06–1.52)], in multiple logistic regression model, only association of single child family with abdominal obesity remained statistically significant [OR: 1.28; 95% CI:1.1–1.50)]. Also in multivariate logistic regression model, for each increase of a child in the family the risk of abdominal obesity [OR: 0.95; 95% CI: 0.91–0.97), high SBP [OR: 0.88; 95% CI: 0.81–0.95)] and generalized obesity [OR: 0.95; 95% CI: 0.91–0.99)] decreased significantly.

**Conclusion:**

The findings of this study serve as confirmatory evidence on the association of cardio-metabolic risk factors with single-child family in children and adolescents. The findings of study could be used for better health planning and more complementary research.

## Background

Over the past decade, the global pattern of diseases has significantly shifted from communicable diseases to the non-communicable diseases (NCDs). This concern mainly rooted in epidemiological transition and rapid changes in lifestyle [1]. Considering the behavioral and biological related risk factors, the backgrounds of childhood NCDs is well documented [2].

More than three-quarters of Cardio Vascular Disease (CVD) deaths occur in low and middle-income countries [3]. Through past three decades, we were witnessing an epidemic of obesity in the world among the children and adolescents [4] has been reported the significant increase in waist circumference (WC), low density lipoprotein (LDL), triglyceride (TG), blood pressure (BP), metabolic syndrome (MetS) and the reduction in high density lipoprotein (HDL) among the adolescents in some countries [5, 6]. In children and teens of developing countries such as Iran and Turkey, it has been shown that the most common factors of MetS are high TG and low HDL [7].

Most of these adverse health outcomes could be prevented by addressing the environmental risk factors such as using tobacco, unhealthy diet and obesity, physical activity, alcohol consumption and harms of using broad population strategies [3].

As another related important point, following the demographic transitions happened in most countries in the world, there has been observed the fertility reduction and changes in family structures [8]. As a result, the numbers of the households have been decreases and the single-child families have been increased [8]. In Iran, such reduction was observed both in urban area and rural areas [9]. The impact of family structure on cardio- metabolic risk factors discussed in many previous attempts. Results of a study showed that, compared to single child, children who are siblings, have more daily physical activity [10, 11]. The association of some of cardio-metabolic risk factors assessed through some scattered studies [12, 13].

Despite the priority of the problem, yet there is an evident gap in the related evidence. Many studies have investigated the association between the cardio- metabolic risk factors and the family structure [14, 15], but due to our knowledge, there is not any research on the association of the cardio- metabolic risk factors and the status of single-child families Therefore, the present study was designed to examine the associations of the single-child family associated with cardio-metabolic risk factors in Iranian children and adolescents.

## Methods

Aim to assess the association between the cardio- metabolic risk factors and the status of single-child family we analyzed the data of comprehensive national survey of CASPIAN-V study was conducted in 2015. Using multistage, stratified cluster sampling method, the study participants selected from, students aged 7–18 years of primary and secondary schools, of urban and rural areas of 30 provinces of Iran. Proportional to size sampling within each province was conducted according to the student’s place of residence (urban or rural) and level of education (primary and secondary) with equal sex ratio. Details on the methodology have been presented before [16], and here we report it in brief.

An expert team of trained health care professionals involved to processes of data gathering. After identifying eligible students, the mission and purpose of the interview was explained. Following informed consent, through interview with students and their parents, specific questionnaires were completed. These questionnaires were extracted from the World Health Organization-Global School Student Health Survey (WHO-GSHS) [17]. Their validity and reliability of Persian-translated questionnaires were confirmed previously [18]. More than demographic information, many aspects of life skills, health behaviors and history of diseases targeted through these questioners [16].

At the next step, by using calibrated instruments, the physical measurements conducted under the standard protocols [17]. During Anthropometric measurements; weight was measured to the nearest 0.1 kg with wearing a light cloth, and height were measured without shoes to the nearest 0.1 cm. Body mass index (BMI) calculated by dividing weight to height squared (m^2^). Using a non-elastic tape, WC was measured at a point midway between the lower border of the rib cage and the iliac crest at the end of normal expiration to the nearest 0.1 cm. Hip circumference was measured, to the nearest 0.1 cm, at the widest part of the hip at the level of the greater trochanter [18].

Blood pressure measured in sitting position, on the right arm, using a mercury sphygmomanometer with an appropriate cuff size. It was measured 2 times at 5-min intervals and the average was registered [19]. BMI categories considered based on he WHO growth curves; to define underweight as age and sex-specific BMI < 5th, overweight as sex-specific BMI for age of 85th -95th, and obesity as sex-specific BMI for >95th [20]. Abdominal obesity was defined as waist-to-height ratio (WHtR) equal to or more than 0.5 [21]. High fasting blood sugar (FBG) ≥ 100 mg/dl, high triglyceride (TG) ≥ 100 mg/dl, high total cholesterol (TC): > 200 mg/dL, high LDL ≥ 110 mg/dl and low HDL <  40 mg/dl (except than15–19-year- old boys< 45 mg/dl) were considered as abnormal [22]. Elevated BP was defined as either high systolic or diastolic BP (SBP/ DBP ≥ 90th percentile for age, sex and height). MetS was defined according to ATP-III criteria modified for children and adolescents [22].

Physical activity (PA) assessed through a validated questionnaire, through which the information of past week frequency of leisure time physical activity outside the school was collected. Enough physical activity was considered as at least 30 min duration of exercises per day that led to sweating and large increases in breathing or heart rate [23].

The Screen time (ST) evaluation of the children was assessed through the questionnaire that contains the average number of hours/day spent on watching T*V*/VCDs, personal computer [24], or electronic games (EG) in time of week days and weekends. The total cumulative spent time categorized into two groups; less than 2 h per day (Low), and 2 h per day or more (High) ( [24]).

Aim to assess the socioeconomic status [25] of students, benefiting from principle component analysis (PCA) method related questions including parental education, parents’ job, possessing private car, school type (public/private), and having personal computer were combined as a unique index values were analyzed as tertiles of low; intermediate and high SES [25].

Underweight, Overweight and obesity in parents were defined according to BMI ≤ 18.5 kg/m2, BMI ≥25 kg/m2 and BMI ≥30 kg/m2, respectively. Abdominal obesity in parents was defined as WC ≥95 cm [26].

### Statistical analysis

Using Stata package ver. 11.0 (Stata Statistical Software: Release 11. College Station, TX: Stata Corp LP. Package), all statistical measures were estimated by survey data analysis methods. Results provide as mean and standard deviation (SD) for continuous variables, and number (percentage) for categorical variables.

Comparing the mean differences between quantitative variables assessed by Student t-test and association between qualitative variables evaluated through the Pearson Chi-square test. Logistic regression analysis considered for evaluation of the association between single-child family and cardio- metabolic risk factors in Iranian children and their families.

For each association three models were run; the first one representing the crude association and in second model additionally association was adjusted for age, living area, sex, physical activity and screen time, SES, family history of obesity. The third model additionally adjusted for BMI in all abnormality except weight disorders. Results of logistic regression revealed as odd ratio (OR) and 95% confidence interval (CI).For all measurements *p*-value of < 0.05 was considered statistically significant.

## Results

**Fig. 1:**
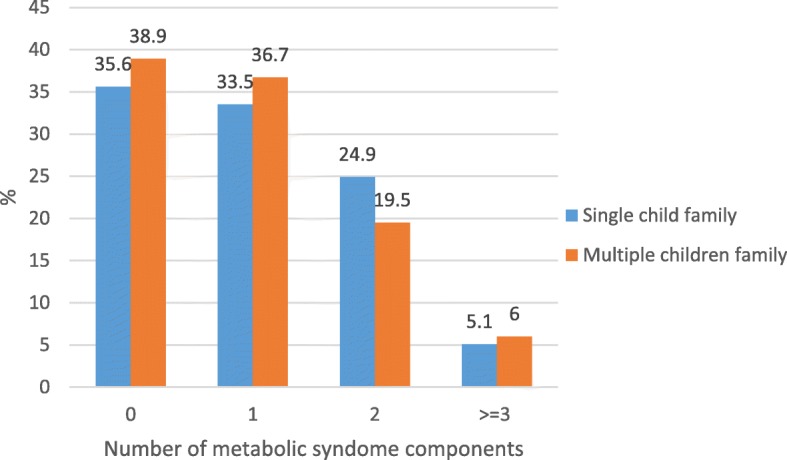
Frequency of metabolic syndrome components according to type of families

Overall, 14,274 students and one of their parents completed the survey (participation rate: 99%). From them 14,151 individuals had complete data for analysis in this study (50.7% boys and 71.4% from urban areas); for blood sampling from students, the participation rate was 91.5% (3843 students out of 4200 students selected for blood sampling). The mean (SD) age of participants was 12.3 (3.2) years with no significant difference between girls and boys. Regarding the distribution of sex and resident area, there was no significant difference between two comparing groups. The mean of height of students in single child families significantly was shorter than the other group [(144.07 ± 18.35) vs. (146.75 ± 17.40), *p* < 0.001] yet the prevalence of abdominal obesity was significantly higher in single child students (26.3% vs. 20.3%, p < 0.001). Given the number of children there was no any detected association between type of families and cardio-metabolic risk factors. Demographic and biochemical characteristics of the participants compared between single/several child families in Table 1. The frequency of MetS components in single child and multiple children families was not statistically different (*P*-value: 0.16) (Fig. 1).

**Table 1:** Demographic and biochemical characteristics of the participants according to single-child family: the CASPIAN V study

Variable		Single child family		
	Total	Yes	No	p-value
Age (year) ^a^	12.28 ± 3.15	11.80 ± 3.24	12.32 ± 3.14	< 0.001
Living area				
Urban ^b^	10,106 (71.4)	822(75.3)	9284(71.1)	0.003
Rural ^b^	4045 (28.6)	270(24.7)	3775(28.9)	
Sex				
Boy ^b^	7172(50.7)	545(49.9)	6627(50.7)	0.595
Girl ^b^	6979(49.3)	547(50.1)	6432(49.3)	
Height (cm) ^a^	147.07 ± 17.53	144.07 ± 18.35	146.75 ± 17.40	< 0.001
Weight (cm) ^a^	41.54 ± 16.93	40.07±16.97	41.5 ± 17.13	0.008
WC (cm) ^a^	66.63 ± 12.1	66.72 ± 12.88	66.71 ± 12.12	0.978
BMI (kg/m^2^) ^a^	18.48 ± 4.69	18.47 ± 4.24	18.51 ± 4.75	0.753
SBP (mmHg) ^a^	98.72 ± 12.91	99.85 ± 13.59	99.10 ± 13.06	0.079
DBP (mmHg) ^a^	63.53 ± 10.19	63.88 ± 10.76	63.83 ± 10.41	0.888
FBG (mg/dL) ^a^	91.66 ± 12.13	92.53 ± 10.00	91.55 ± 12.28	0.119
TG (mg/dL) ^a^	88.16 ± 45.27	90.74 ± 42.05	87.88 ± 45.49	0.272
HDL-C (mg/dL) ^a^	46.16 ± 9.98	46.32 ± 11.25	46.16 ± 9.86	0.822
LDL-C (mg/dL) ^a^	90.06 ± 22.64	90.57 ± 24.06	90.02 ± 22.47	0.710
TC (mg/dL) ^a^	153.85 ± 27.47	155.04 ± 28.93	153.76 ± 27.27	0.472
Physical activity				
Low ^b^	8160 (58.2)	696(64.4)	7464(57.7)	< 0.001
High ^b^	5859 (41.8)	385(35.6)	5474(42.3)	
Screen Time				
Low ^b^	13,067(92.5)	979(90.0)	12,088(92.7)	0.001
High ^b^	1065(7.5)	109(10.0)	956(7.3)	
SES				
Low ^b^	4496 (33.2)	168(16.9)	4328(34.5)	< 0.001
Medium ^b^	4510 (33.3)	304(30.5)	4206(33.5)	
High ^b^	4544 (33.5)	525(52.7)	4019(32.0)	
Abdominal obesity ^b^	2950 (21.1)	283(26.3%)	2667(20.6)	< 0.001
High SBP ^b^	433 (3.1)	50(4.7)	383(3.0)	0.002
High DBP ^b^	1436 (10.3)	121(11.3)	1315(10.3)	0.293
High BP ^b^	1589 (11.4)	143(13.3)	1446(11.3)	0.043
High FBG ^b^	161 (4.2)	8(2.8)	153(4.3)	0.219
High TG ^b^	1060 (27.7)	87(30.5)	973(27.5)	0.275
Low HDL-c ^b^	1127 (29.5)	86(30.2)	1041(29.4)	0.793
High TC ^b^	187(4.9)	15(5.3)	172(4.9)	0.764
High LDL ^b^	670(17.5)	58(20.4)	612(17.3)	0.194
MetS ^b^	188 (5.1)	17(6.0)	171(5.0)	0.435
Weight status				
Underweight ^b^	2270(16.2)	144(13.4)	2126(16.4)	0.008
Normal weight ^b^	8819(62.9)	685(63.8)	8134(62.8)	
Overweight ^b^	1321(9.4)	96(8.9)	1225(9.5)	
Obesity ^b^	1606(11.5)	149(13.9)	1457(11.3)	0.010

Overweight: BMI; 85th–95th; obesity, BMI > 95th; low HDL: < 40 mg/dL (except in boys 15–19 y old, that cut-off was < 45 mg/dL); high LDL: > 110 mg/dL; high TG: 150 mg/dL; high TC: > 200 mg/dL; elevated FBS > 100 mg/dL; high blood pressure: > 90th (adjusted by age, sex, height); MetS: ATP-III criteria

*SES* socioeconomic status, *SBP* systolic blood pressure, *DBP* diastolic blood pressure, *BP* blood pressure, *FBG* fasting blood glucose, *TG* triglycerides, *HDL* high density lipoprotein, *LDL* low density lipoprotein, *TC* total cholesterol, *MetS* metabolic syndrome, *BMI* body mass index, *WC* waist circumference

^a^ Data are presented as mean ± standard deviation

^b^ Data are presented as number (%)

Comparing the characteristics of the two groups of study, no significant difference was found between age and anthropometric indices of mothers and fathers of single/several child families (Table 2).

**Table 2:** Parental characteristics of participants according to single child family: the CASPIAN V study

Variable		Single child family		p-value
	Total	Yes	No	
Mother				
WC (cm) ^a^	87.75 ± 14.29	86.90 ± 14.56	87.78 ± 14.21	0.076
BMI (kg/m^2^) ^a^	26.74 ± 5.02	26.52 ± 4.65	26.75 ± 5.05	0.143
Age (year) ^a^	38.11 ± 6.45	36.33 ± 6.83	38.25 ± 6.37	< 0.001
Weight status				
Underweight ^b^	451(4.0)	14(1.5)	437(4.2)	< 0.001
Normal weight ^b^	3951(34.7)	387(42.0)	3564(34.1)	
Overweight ^b^	4224(37.1)	316(34.3)	3908(37.4)	
Obesity ^b^	2749(24.2)	204(22.1)	2545(24.3)	
abdominal obesity ^b^	3446(30.3)	245(26.4)	3201(30.70)	0.007
Father				
WC (cm) ^a^	87.03 ± 16.65	89.20 ± 17.96	86.97 ± 16.49	0.162
BMI (kg/m^2^) ^a^	25.11 ± 4.07	25.66 ± 4.35	25.08 ± 4.05	0.135
Age (year) ^a^	44.19 ± 7.05	41.96 ± 6.74	44.25 ± 7.01	< 0.001
Weight status				
Underweight ^b^	172(6.8)	3(2.2)	169(7.1)	0.011
Normal weight ^b^	1032(40.7)	64(46.4)	968(40.4)	
Overweight ^b^	1062(41.9)	49(35.5)	1013(42.3)	
Obesity ^b^	269(10.6)	22(15.9)	247(10.3)	
Abdominal obesity ^b^	886(35.5)	50(37.0)	836(35.4)	0.695

Parental underweight: BMI ≤18.5 kg/m^2^; Parental overweight: BMI ≥25 kg/m^2^; parental general obesity: BMI ≥30 kg/m^2^; parental abdominal obesity: waist circumference ≥ 95 cm

*BMI* body mass index, *WC* waist circumference

^a^Data are presented as mean ± standard deviation

^b^Data are presented as number (%)

Although in univariate logistic regression model (Model I), single child students had an increased risk of abdominal obesity [OR: 1.37; 95% CI: 1.19–1.58)], high SBP [OR: 1.58; 95% CI:1.17–2.14)], high BP [OR: 1.21; 95% CI:1.01–1.45)] and generalized obesity [OR: 1.27; 95% CI:1.06–1.52)], in multiple logistic regression model, only association of single child family with abdominal obesity remained statistically significant [OR: 1.28; 95% CI:1.1–1.50)].

In multivariate logistic regression model, for every increase of a child in the family the risk of abdominal obesity [OR: 0.95; 95% CI: 0.91–0.97), high SBP [OR: 0.88; 95% CI: 0.81–0.95)] and generalized obesity [OR: 0.95; 95% CI: 0.91–0.99)], decreased significantly (Table 3).

**Table 3:** Association of Single child family with cardio-metabolic risk factors in logistic regression analysis: the CASPIAN V study

Variable	Single child family (yes/ no)		Number of children	
	OR	95% CI	OR	95% CI
Abdominal obesity				
Model I	1.37	1.19–1.58*	0.93	0.90–0.95*
Model II	1.28	1.1–1.50*	0.94	0.91–0.97*
High SBP				
Model I	1.58	1.17–2.14*	0.87	0.81–0.93*
Model II	1.35	0.96–1.90	0.88	0.81–0.95*
Model III	1.34	0.95–1.90	0.88	0.81–0.95*
High DBP				
Model I	1.11	0.91–1.35	1.00	0.96–1.03
Model II	1.04	0.83–1.30	1.00	0.96–1.05
Model III	1.02	0.81–1.28	1.01	0.97–1.06
High BP				
Model I	1.21	1.01–1.45*	0.98	0.94–1.01
Model II	1.15	0.93–1.41	0.98	0.94–1.02
Model III	1.13	0.92–1.39	0.98	0.94–1.03
High TG				
Model I	1.15	0.89–1.50	1.02	0.98–1.07
Model II	1.3	0.97–1.72	1.02	0.97–1.08
Model III	1.31	0.98–1.74	1.03	0.97–1.08
Lowe HDL-c				
Model I	1.03	0.79–1.34	1.02	0.98–1.06
Model II	1.16	0.87–1.56	0.99	0.93–1.04
Model III	1.17	0.87–1.57	0.99	0.94–1.04
High FBG				
Model I	0.63	0.31–1.31	0.98	0.89–1.08
Model II	0.51	0.22–1.19	1.04	0.92–1.18
Model III	0.51	0.22–1.18	1.04	0.92–1.18
MetS				
Model I	1.22	0.73–2.05	0.96	0.88–1.06
Model II	1.14	0.63–2.06	0.94	0.84–1.06
Model III	1.08	0.59–1.99	0.95	0.84–1.06
High LDL-c				
Model I	1.22	0.90–1.65	1.03	0.98–1.08
Model II	1.29	0.93–1.79	1.03	0.97–1.10
Model III	1.30	0.93–1.80	1.03	0.97–1.10
High TC				
Model I	1.08	0.63–1.86	0.93	0.85–1.03
Model II	0.97	0.53–1.75	0.97	0.86–1.09
Model III	0.99	0.54–1.79	0.98	0.87–1.10
Overweight				
Model I	0.94	0.75–1.16	0.97	0.94–1.01
Model II	0.86	0.68–1.10	0.98	0.94–1.03
Obesity				
Model I	1.27	1.06–1.52*	0.91	0.88–0.94*
Model II	1.15	0.94–1.41	0.95	0.91–0.99*

Model I: without adjustment

Model II: adjusted for age, living area, sex, physical activity and screen time, SES, family history of obesity

Model III: additionally adjusted for BMI in all abnormality except weight disorders

Overweight: BMI; 85th–95th; obesity, BMI > 95th; excess weight, BMI > 85th; low HDL: < 40 mg/dL (except in boys 15–19 y old, that cut-off was < 45 mg/dL); high LDL: > 110 mg/dL; high TG: 150 mg/dL; high TC: > 200 mg/dL; elevated FBS > 100 mg/dL; high blood pressure: > 90th (adjusted by age, sex, height); MetS: ATP-III criteria;

*SBP* systolic blood pressure, *DBP* diastolic blood pressure, *BP* blood pressure, *FBG* fasting blood glucose, *TG* triglycerides, *HDL* high density lipoprotein, *LDL* low density lipoprotein, *TC* total cholesterol, *MetS* metabolic syndrome

^*^ *p*-value ˂ 0.05

## Discussion

Based on our knowledge this is the first investigation on the association between the single-child and cardio-metabolic risk factors in national representative data. The results of study have shown that there is a significant statistical association between the single-child family and the obesity among children and adolescents. It is considerable that, there was not significant association between the single-child and other cardio-metabolic risk factors.

There is some evidence on the family structure and its association with the NCDs or their cores pound risk factors. The association of single child dimension of family with increased risk of obesity have been confirmed in previous investigations [27]. In another study, it has been shown that, compared to the single children, the students who have a sister or a brother are less likely to be obese [12]. Another research shown the association between more siblings and less risk for obesity [28].

In the logistic model, there is no significant association between the dimension of family and the risks of high SBP, high DBP, high BP, high TG, low HDL-c, high FBS, MetS, high LDL-c and high TC. When we run the linear model, we investigate the significant association between the numbers of children the decreased risk of high SBP (OR: 0.88, CI: 0.81, 0.95).

Based on the evidence; boys in single-child families, compare with their counterpart in numerous child families; significantly spent more time for watching TV [29] and less time for physical activities. The physical activities shown the inverse association with the levels of LDL and TC [30]. Increasing screen time during a week with, discussed as a predisposing factor of obesity, overweight, diabetes, CVD and MetS [31–33].

There is some discussion that shown single children because their sense of loneliness, mostly spend more time for watching TV. This face them with increased risk of cardiometabolic risk factors. Consumption junk food is one of the probable related factors for insulin resistance and high risks of SBP [34]. On the other hand, junk food intake is positively associated with levels of BMI, WC, and TG level [35].

Studies shown that, smaller size families mostly demand for processed outdoors foods. Such nutritional habits could increase the levels of TG and the risk of cardio- metabolic diseases [36]. Some studies also emphasized on the link of fast food consumption with increased levels of serum fat and calorie intake in children obesity [37].

However, we could not found any significant association between single child situation and the majority of metabolic cardiovascular risk factors, but the role of a healthy lifestyle including physical activity and nutrition in cardio- metabolic risk factors emphasized.

One of the strengths of the present study was its national representative large sample of children and adolescents. Considering the nature of study design, the cross-sectional study limit us in causality inference of variables. On the other hand recalling bias should be mentioned as another limitation.

## Conclusion

The findings of present study provide the confirmatory evidence on the association of cardio-metabolic risk factors with single-child family in national sample of children and adolescents. As a considerable point the mean of height of students in single child families significantly was shorter than the other group. The findings of study could be used for better health planning and more evidence-based policy making. The achievements also highlighted the path of complementary research.

## Abbreviations

BMI: Body Mass Index
BP: blood pressure
CI: Confidence Interval
CVD: Cardio Vascular Disease
DBP: diastolic blood pressure
EG: Electronic Games
FBG: fasting blood glucose
HDL: high density lipoprotein
LDL: low density lipoprotein
MetS: metabolic syndrome
NCDs: Non-Communicable Diseases
OR: Odd Ratio
PA: Physical Activity
PCA: Principle Component Analysis
SBP: systolic blood pressure
SD: Standard Deviation
SES: socioeconomic status
ST: Screen Time
TC: total cholesterol
TFR: Total Fertility Rate
TG: triglycerides
WC: Waist Circumference
WHO-GSHS: World Health Organization-Global School Student Health Survey
WHtR: Waist-to-Height Ratio
